# The Impact of Mobile Applications on Improving Oral Hygiene Knowledge and Skills of Adolescents: A Scoping Review

**DOI:** 10.3390/jcm14092907

**Published:** 2025-04-23

**Authors:** Alice Murariu, Livia Bobu, Gabriela Luminița Gelețu, Simona Stoleriu, Gianina Iovan, Roxana-Ionela Vasluianu, Cezar Ilie Foia, Diana Zapodeanu, Elena-Raluca Baciu

**Affiliations:** 1 Department of Surgicals, Faculty of Dental Medicine, “Grigore T. Popa” University of Medicine and Pharmacy, 700115 Iasi, Romania; alice.murariu@umfiasi.ro (A.M.); gabriela.geletu@umfiasi.ro (G.L.G.); zapodeanu.diana@d.umfiasi.ro (D.Z.); 2 Department of Cariology, Faculty of Dental Medicine, “Grigore T. Popa” University of Medicine and Pharmacy, 700115 Iasi, Romania; simona.stoleriu@umfiasi.ro (S.S.); gianina.iovan@umfiasi.ro (G.I.); 3 Department of Implantology, Removable Prostheses, Dental Technology, Faculty of Dental Medicine, “Grigore T. Popa” University of Medicine and Pharmacy, 700115 Iasi, Romania; roxana.vasluianu@umfiasi.ro (R.-I.V.); elena.baciu@umfiasi.ro (E.-R.B.); 4 Department of Orthopedics—Traumatology, Faculty of Medicine, “Grigore T. Popa” University of Medicine and Pharmacy, 700115 Iasi, Romania

**Keywords:** adolescents, mHealth, mobile applications, knowledge, skills, oral hygiene behavior, dentistry

## Abstract

**Background/Objectives**: During adolescence, dental caries, erosions, and gingival diseases can significantly impact quality of life. Currently, mobile applications are widely used in health promotion, especially among teenagers, as they offer a modern approach to oral health education. This scoping review aims to evaluate the effectiveness of mobile health (mHealth) applications in improving adolescents’ behavior and knowledge regarding oral hygiene. **Methods**: Searches were conducted in the Medline/PubMed, Scopus, Web of Science, Embase, and Google Scholar databases. Both randomized and non-randomized clinical trials published between 2015 and 2024 were analyzed. Selected studies evaluated oral hygiene behavior (knowledge and skills), as well as Plaque Index and gingival health indicators, by comparing a control group receiving traditional education with an intervention group using mobile applications for education. **Results**: Out of 738 articles found, only 21 met the eligibility criteria. Of the total number of included studies, 14 (66.6%) reported that adolescents in the intervention group utilizing mobile applications demonstrated superior plaque control and reduced gingival bleeding compared to those in the control group. Notably, this outcome was observed even in studies employing a single-group design. In contrast, five studies (23.8%) found no statistically significant differences between groups, while two studies (9.6%) indicated that traditional educational methods were more effective than modern methods. **Conclusions**: The analyzed studies indicate that mobile health applications can be valuable tools for improving adolescents’ oral health. However, some findings also demonstrate that traditional education methods yield similar positive effects.

## 1. Introduction

Oral diseases affected 3.5 billion people worldwide in 2019, and their incidence is increasing, especially in low- and middle-income countries [1]. According to the latest World Health Organization (WHO) report, in 2019, the prevalence of permanent tooth decay was 29%, increasing by 640 million cases between 1990 and 2019 [1]. Untreated cavities cause pain, discomfort, reduced quality of life, lower self-esteem, and impaired social life [2].

In this context, concrete measures to reduce the incidence of dental caries are essential. These measures include the implementation of preventive and educational programs for oral health, targeting vulnerable age groups, such as preschool children and adolescents [3,4,5], mainly aiming to reduce risk factors associated with the development of carious lesions. The messages conveyed during these educational lessons focus on reducing sugar consumption, improving oral hygiene, using fluoride, and ensuring regular dental check-ups.

A variety of strategies, methods, and tools can be used for oral health education. The forms of health education can be auditory (individual or group conversations, conferences, symposiums, lectures, lessons, seminars, or Q&A sessions); visual (flyers, leaflets, brochures, posters, charts, diagrams, drawings, caricatures, or models); audiovisual (movies, cartoons, TV programs, or theater, such as drama or puppet shows); or complex (exhibitions, trips, book stands, contests, or practical demonstrations).

One of the key factors in choosing the appropriate form of oral health education is the age of the individual or target population.

Adolescence is defined as the age range from 10 to 19 years, a period of multiple biological, emotional, and behavioral changes that impact general and oral health. It is also a crucial stage for acquiring knowledge, skills, relationship management, and other essential attributes necessary for a successful transition to adulthood [6,7,8].

Many risk behaviors that begin in adolescence, such as smoking, alcohol consumption, and drug use, can be prevented through appropriate measures. For adolescents, adherence to preventive measures represents a challenge, as immediate benefits are difficult to understand, and long-term outcomes are not fully perceived [9,10]. Although traditional education has proven effective in changing health behaviors, traditional approaches may pose challenges related to the need for specialists, logistics, or maintaining individual motivation over a long period. In this regard, digital technology, with its characteristics (wide reach, low cost, and attractiveness), can offer an alternative to traditional education [11,12].

Recently, a rapid increase in the number of mobile phones has been observed, which have become an integral part of people’s lives, successfully used in many fields, including health, education, and entertainment [13]. Currently, the number of mobile phones is rising, with approximately 4.88 billion smartphones worldwide in 2024, covering about 60.42% of the global population [14].

According to the WHO report from mid-2020, 96.7% of the world’s population lived within the coverage of a mobile network. Additionally, a higher number of mobile connections than individuals was found worldwide, with 105 subscriptions per 100 inhabitants in 2020 [15].

There are many advantages to using applications in healthcare, such as self-monitoring of behavior, adherence to medications and treatments, increased motivation, disease prevention and management, doctor–patient communication, action planning, and behavioral goal review [16]. With digital technology, remote consultations (teledentistry) can be conducted, motivational messages can be sent to improve treatment adherence, and various mobile applications (mHealth) can be accessed via smartphones.

Mobile health (mHealth) refers to the use of mobile technologies for health interventions [15]. Mobile phones, tablets, and other mobile and digital technologies are used to provide information, training, and access to health services. There is growing evidence supporting the use of mHealth through tools such as text messaging, video messaging, phone calls, mobile internet, mobile applications, visualization and diagnostic tools, and monitoring devices [17].

Although mHealth applications have existed for years, the mobile health app market grew significantly after the outbreak of COVID-19. In 2020, the healthcare sector experienced significant growth in the use of applications, with many companies marketing apps that provide diagnostics and connect patients with online healthcare providers [18].

In 2021, 44% of U.S. consumers used digital tools to track their health, and 33% owned a wearable health or wellness device, with the global mHealth app market expected to reach USD 105.9 billion by 2030 [19].

In dentistry, mobile applications are used in almost all areas, including preventing dental caries [20,21,22,23,24] and periodontal disease [25,26], detecting suspicious oral lesions [27] and traumatic injuries [28], motivating orthodontic patients [29], detecting carious lesions [30,31], oral hygiene education [32], helping anxious patients [33], raising parental awareness about their children’s oral health [34,35], and improving oral hygiene in older adult patients [36]. Moreover, dental erosion is frequently encountered in adolescents, which necessitates both rigorous oral hygiene and non-invasive remineralization treatment. Among the most advanced non-invasive options are toothpastes containing zinc carbonate hydroxyapatite, which have shown effectiveness within a short period in pediatric patients, as demonstrated by a randomized clinical study conducted by Scribante et al. [37]. To further support the management of dental erosion, a dedicated application called Intact-Tooth [38] has been developed, providing valuable resources for both patients and dental professionals.

It is widely recognized that digital methods are preferred by adolescents and young people over traditional educational methods, as they tend to favor short text messages (SMS), multimedia messages (MMS), or videos over brochures or pamphlets [39,40]. Moreover, unlike traditional approaches, mobile education allows for greater accessibility, enabling frequent and sustained contact with health information, offering a fun, interactive, and personalized platform for improving oral health [41].

However, the effectiveness of digital methods in improving oral hygiene knowledge, attitudes, behaviors, and, consequently, oral health indicators in adolescents is not clearly established in the literature. In recent years, review-type studies have been conducted assessing app-based oral health promotion interventions on modifiable risk factors associated with early childhood caries, addressed to parents/caregivers [42]; mobile health applications for children’s oral health improvement [43]; oral hygiene improvement in orthodontic patients [16]; gamification and its impact on oral health of children and adolescents [18,44,45]; mobile apps for dental caries prevention [46]; and the effect of mHealth in improving oral hygiene of adolescents and adults [32,40], but no reviews have focused on teenagers. In this context, the purpose of this review is to address this gap in knowledge by systematizing the results of existing research in this field and identifying the need for future research directions. The data obtained from this review could be useful both for practical application and research, contributing to the development of mHealth strategies for improving the oral health of teenagers.

## 2. Materials and Methods

This scoping review followed the methodology developed by Aromataris and collaborators in the JBI Manual of Evidence Synthesis [47]. It was drafted using the Preferred Reporting Items for Systematic Reviews and Meta-Analyses (PRISMA) framework, with the PRISMA extension for scoping reviews (Table S1—Supplementary Material) [48], to ensure a comprehensive and systematic approach.

The key stages of the research included defining the research question, developing a comprehensive search strategy, establishing inclusion/exclusion criteria for studies, selecting and analyzing studies based on these criteria, extracting relevant information, and synthesizing the obtained results.

### 2.1. Review Questions

The research aimed to answer the following questions: (1) Are mobile applications effective in improving adolescents’ oral hygiene? and (2) Are these applications more useful and effective compared to traditional oral health education methods?

These questions were formulated to guide the review process in achieving the proposed objectives of the research.

### 2.2. Information Sources and Search Strategies

A systematic search was conducted using keywords in the PubMed, Scopus, Web of Science, Embase, and Google Scholar databases, covering the period from 2015 to 2024. The selected databases were searched to identify relevant articles on oral hygiene, adolescents, and mobile health.

To ensure the inclusion of all relevant articles in the review, the search strategy involved combining the keywords “teledentistry”, “mHealth”, “mobile application”, “adolescents”, “oral hygiene”, “oral health”, and “oral health promotion” using the Boolean operators AND and OR, allowing for a refined search. The search strategies are described in Table 1.

### 2.3. Inclusion and Exclusion Criteria

To ensure the quality and relevance of the articles included in this scoping review, a set of specific inclusion and exclusion criteria was established (Table 2).

The review included full-text articles published in English between 2015 and 2024, focusing on randomized and non-randomized clinical trials conducted among adolescents (10–19 years old). The selected studies analyzed the use of mobile health (mHealth) technology for raising awareness of the importance of oral hygiene in preventing dental caries and gingival diseases.

Conversely, the review excluded articles published before 2015, abstract-only publications, articles published in languages other than English, studies conducted on age groups outside the 10–19 range (children under 10 or adults over 19), systematic reviews, narrative reviews, scoping reviews, qualitative studies (e.g., focus groups), and irrelevant articles that did not align with the study’s objectives.

The article selection process was carried out in two stages. In the first stage, the titles and abstracts of the retrieved articles were screened for relevance based on the inclusion and exclusion criteria. Subsequently, two independent reviewers (A.M. and L.B.) assessed the available full-text articles to determine their eligibility according to the predefined inclusion and exclusion criteria. Any differences of opinion among the reviewers during each stage of the selection process were resolved with the help of an additional evaluator (E.-R.B.).

The data extracted from the selected articles were entered into a specially designed data extraction form. This form included details such as the first author of the article, year of publication, country where the research was conducted, study objective, study sample (number of subjects, age, specific characteristics, and any orthodontic treatments), intervention performed, type of mHealth technology used, clinical parameters assessed, and the main findings of the study.

By synthesizing the obtained data, the review aimed to provide a clearer picture of the effectiveness of mobile applications in improving adolescents’ oral hygiene behavior and knowledge, as well as to highlight any uncertainties or gaps in research in this field and to outline future research directions. 

### 2.4. Risk of Bias Assessment

To evaluate the risk of bias in randomized clinical trials, the Cochrane Risk of Bias 2 (RoB 2) tool was employed. The five criteria covered by this tool are bias arising from the randomization process, deviations from intended interventions, missing outcome data, measurement of the outcome, and selection of the reported result [49]. For non-randomized clinical trials, the Risk Of Bias In Non-randomized Studies—of Interventions (ROBINS-I) tool was applied. The assessments were independently performed by two reviewers (A.M. and L.B.), with any disagreements resolved through discussion and, when necessary, by consultation with a third reviewer (E.-R.B.).

## 3. Results

**Figure 1:**
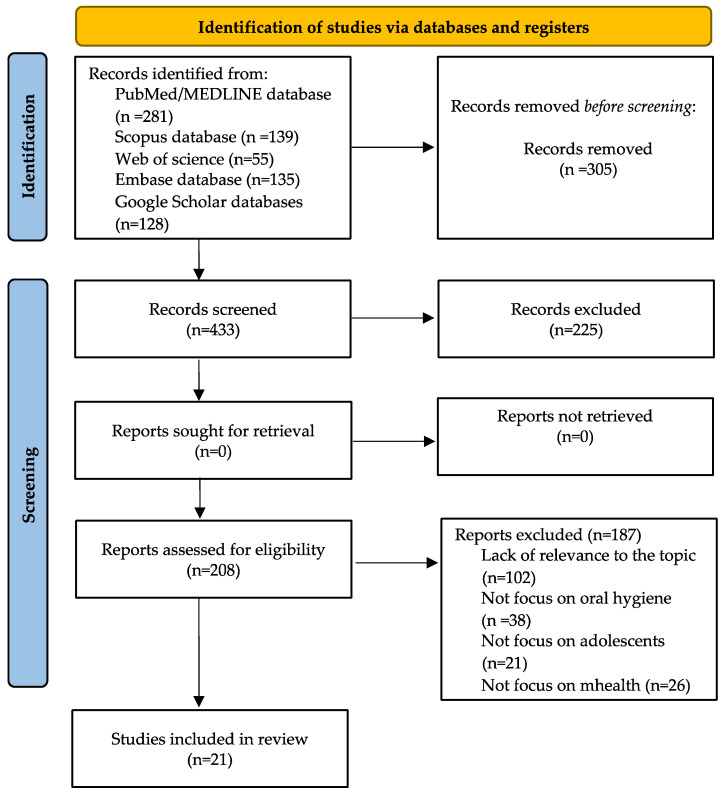
The flow diagram for the identification, screening, and eligibility of studies (PRISMA-ScR).

A total of 738 articles were retrieved between December 2024 and January 2025 from the following databases: PubMed/MEDLINE (281 articles), Scopus (139 articles), Web of Science (55 articles), Embase (135 articles), and Google Scholar (128 articles). Of these, 305 were excluded due to duplication, availability only as abstracts, or lack of accessibility. From the remaining 433 records, 225 were deemed irrelevant, leaving 208 articles for eligibility assessment. Among these, 187 articles were excluded due to the following reasons: lack of relevance to the topic, not focusing on oral hygiene, not focusing on adolescents, or not focusing on mHealth. Ultimately, 21 articles were included in the analysis. The search results and study inclusion process are presented in a PRISMA-ScR (Preferred Reporting Items for Systematic Reviews and Meta-Analyses extension for Scoping Reviews) flow diagram in Figure 1 [48].

### 3.1. General Characteristics of the Selected Articles

Out of the 21 selected studies, 18 were randomized controlled clinical trials [50,51,52,53,54,55,56,57,58,59,60,61,62,63,64,65,66,67] and 3 were non-controlled interventional studies [68,69,70], including 1 study conducted without a control group [70].

From the selected clinical trial studies, we found that most researchers included two groups of adolescents: a control group that received traditional education and an intervention group that received educational messages or benefited from mobile phone applications. Furthermore, clinical evaluations were performed, such as the amount of dental plaque assessed using the Plaque Index (PI), gingival health measured by the Gingival Index (GI), and the presence of early carious lesions known as white spot lesions (WSLs). These parameters were assessed at the beginning of the study and at various times, typically at 6 months and 12 months. Additionally, seven studies specifically targeted adolescents with fixed orthodontic appliances [55,56,57,59,62,66,67].

Some studies included a questionnaire to assess both oral health knowledge and practices [54,59,61,63,64,66,68], while others used the questionnaire solely to evaluate the level of knowledge regarding oral hygiene [51,58,70].

The extracted and collected data from the included articles are detailed in Table 3 and organized according to the following parameters: author, year, country, purpose, study design, sample size, test and control group, follow-up time, and findings.

### 3.2. Distribution of Articles by Year of Publication

**Figure 2:**
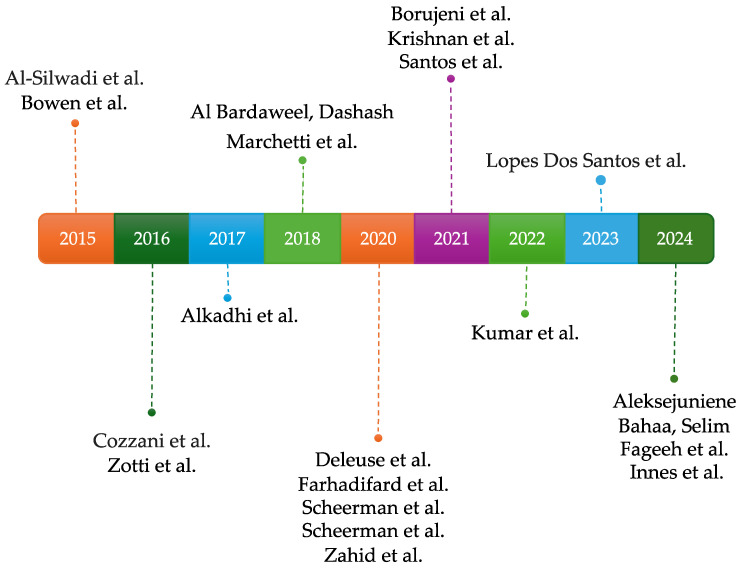
The distribution of articles by publication year [50,51,52,53,54,55,56,57,58,59,60,61,62,63,64,65,66,67,68,69,70].

As illustrated in Figure 2, the distribution of articles spans the period from 2015 to 2024. The highest numbers of studies were published in 2020 and 2024, indicating peak research activity in those years, while the lowest numbers were recorded in 2017, 2022, and 2023, with only one article published in each of these years.

### 3.3. Distribution of Studies by Type of Technology Used

**Figure 3:**
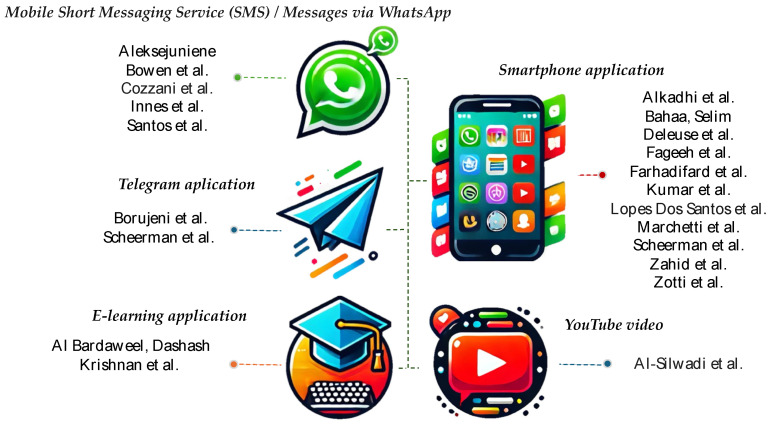
The distribution of studies by the mobile technology used [50,51,52,53,54,55,56,57,58,59,60,61,62,63,64,65,66,67,68,69,70].

Figure 3 shows that the most frequently used applications in the selected studies were mobile smartphone applications (11), followed by text messages on WhatsApp (5), e-learning (2), and the Telegram application (2), while the YouTube platform was used in only one study.

### 3.4. Risk of Bias in Included Studies

As shown in Table 4, out of the 18 articles, only 6 studies provided high-quality evidence (low risk), evidence was a concern for 5 studies, and the remaining 7 studies presented low-quality evidence (high risk).

For the three non-randomized controlled trials included in the review (Table 5), the ROBINS-I assessment indicated a risk of bias: a serious risk of bias in the study by Aleksejuniene, a moderate risk in the study by Krishnan, and a low risk in the study by Zahid.

## 4. Discussion

This systematic review provides an overview of mobile application approaches used in promoting oral health among adolescents, particularly for improving oral hygiene. Mobile applications installed on smartphones enable the technological development of user-adapted software to reflect their specific needs, allowing for responsive, confidential, and targeted communication channels [71]. Mobile applications are widely accepted by children and adolescents and offer new opportunities to engage them in personal oral health care. They are considered a more advantageous method compared to traditional approaches for improving healthy behaviors, education, and health monitoring [72].

The age range of 10–19 years is a crucial period for developing healthy behaviors and avoiding unhealthy ones through interventions that can motivate patients to maintain and care for their oral health. This plays a key role in preventing dental caries and periodontal diseases, emphasizing the need for efforts to improve oral hygiene through various methods.

In orthodontic treatments, poor oral hygiene can negatively affect treatment outcomes [55]. Gingival inflammation has harmful effects on the periodontium, including recession, hyperplasia, and subsequent periodontal disease. Orthodontic treatment with fixed appliances increases the risk of enamel demineralization, which is exacerbated in patients with poor oral hygiene. Effective plaque removal and adherence to oral hygiene are major concerns for orthodontists, leading to numerous interventional studies conducted among their patients [50,51,57,59,62,63,64,67].

Currently, numerous mobile applications exist that are designed to help patients improve their behavior for maintaining strict oral hygiene. For example, in 2021, Chen and colleagues reported in Australia the existence of 562 applications in the Google Play Store and iTunes, of which 40 focused on preventing dental caries and periodontal diseases. Among these, oral hygiene was the most frequently targeted preventive behavior, addressed in 93% of applications, while dietary intake was covered in 45%, and fluoride use was included in 42% of applications [46]. For this reason, most of the selected studies (11) used mobile phone applications, followed by text messages and social networks such as Telegram, e-learning platforms, and other digital platforms like YouTube, which are frequently used by adolescents and young people.

After analyzing the selected studies, we found that the research results were not uniform, leading to several different observations.

The majority of studies (n = 11) reported that participants in the intervention groups achieved statistically significantly better outcomes in oral hygiene compared to those in the control groups [51,53,54,55,56,58,59,63,65,66,67]. Additionally, six studies documented notable reductions in Plaque Index and Gingival Index values following the use of mobile health interventions [50,54,61,63,64,65]. Furthermore, a non-randomized study without a control group conducted by Aleksejuniene [68] demonstrated improvements in toothbrushing skills and duration, as well as increased dietary knowledge, after participants received oral health education through mobile phone text messages.

However, other authors, such as Deleuse [57] and Krisnan [69], observed decreases in PI and GI values in both study groups, without statistically significant differences.

Finally, a third group of studies indicated no statistically significant differences between the control and intervention groups regarding improvements in gingival health and plaque levels. This conclusion was reported by Innes [60], Lopes de Santos [62], and Zahid [70]. Moreover, Zahid [70] noted that using mobile applications was more challenging than traditional health education lessons. A similar conclusion was reached by Al Bardaweel [52] in a study comparing adolescents who received education through leaflets and those who received e-learning education: the leaflet cluster had statistically significantly better oral health knowledge than the e-learning cluster at 6 weeks (*p* < 0.05) and at 12 weeks (*p* < 0.05).

Regarding the incidence of white spot lesions, Zotti [67] observed a reduction in their occurrence among patients in the intervention group wearing fixed multibracket appliances. In contrast, Deleuse [57], in a similar category of orthodontic patients, found that the incidence remained constant in both intervention and control groups.

Sheerman [66] adopted the concept of the two previously mentioned research groups (adolescents receiving traditional education vs. those receiving education through mobile applications) but introduced a third group including adolescents and their mothers. He found that young people in the third group exhibited better oral hygiene behavior and had lower plaque and gingival bleeding indices compared to those in the intervention and control groups. Additionally, it was noted that “the adolescents in the mother-and-adolescent group reported significantly more perceived social support from their mothers compared to those in the intervention and control groups at six months of follow-up”.

In another study conducted in Canada, Aleksejuniene [68] incorporated a dietary knowledge assessment questionnaire in which adolescents evaluated each food or drink as “tooth-friendly” or not. The study also examined the consumption of vegetables/fruits, carbonated drinks, juices, and sugar-containing products. Oral health messages were sent to all participants via SMS twice a week for one month. Evaluations were conducted after 4–5 weeks, assessing oral self-care skills and total brushing duration (in seconds). Over time, improvements were observed in oral hygiene skills, an increase in toothbrushing time, better knowledge of dietary hygiene, and a reduction in sugar consumption.

A special section of research focused on adolescents with autism spectrum disorder (ASD) and those affected by disabilities (visual or hearing) [58,69].

Krishnan [69] tested the effectiveness of a mobile application (Brush Up) in a sample of 60 adolescents with autism, divided into a control group and an intervention group. Their Plaque Index and Gingival Index were assessed at the beginning of the study and again at 6 and 12 weeks. The results showed no statistically significant differences in the clinical indices evaluated between the two groups.

Fageeh [58] conducted an oral health education program for 50 adolescents with blindness and 50 adolescents with deafness using an innovative platform called Telesmile, adapted specifically for each group. Participants were divided into two groups: the control group received traditional oral hygiene instructions, while the intervention group received guidance through the mobile application. Their knowledge was assessed at the initial visit and again after four weeks. The participants’ oral hygiene knowledge was very poor at the beginning of the study but significantly improved (*p* < 0.001) after four weeks with the help of the Telesmile application.

Mobile applications have proven to be useful in improving oral hygiene in adults, too. Review studies focusing on adult age groups and those including subjects from all age groups have not highlighted differences between the efficiency of using these applications in adults and in adolescents. However, older adults might require individualized oral health information to feel motivated to adopt mHealth oral health knowledge [73,74,75], while adolescents show greater ease in using new technologies. The longer-term maintenance of positive results remains to be assessed through longitudinal studies.

As a result of the conducted analysis, we can conclude that mHealth applications, combined with messaging and social media, can represent a novel oral health strategy that provides adolescents with timely, accurate, and engaging education, improving behaviors and achieving better gingival and dental health. This is evidenced by a reduction in early white spot lesions, decreased plaque accumulation, and reduced gingival bleeding.

Based on the review of selected articles, we can confidently answer our initial questions: mobile applications represent an attractive educational tool for adolescents, proving to be useful and, in some cases, even more effective than traditional oral health education methods.

### Gaps in Literature and Future Research Challenges

The variability of the observed effects across studies may be caused by differences in behaviors, the educational and cultural backgrounds of adolescents’ families, and the heterogeneity of intervention approaches.

However, further research is needed on these intervention strategies, using rigorous study designs on large population samples to evaluate the effectiveness and cost-efficiency of mobile applications in promoting preventive behaviors among adolescents. Since most studies included in this review had short follow-up periods, future research should extend over longer durations to assess whether patient motivation remains consistent or declines over time. Additionally, the availability of such studies would allow for a more comprehensive assessment, such as a systematic review and meta-analysis, to provide a clearer picture of the effectiveness of these interventions.

Finally, the challenges associated with using mobile applications in dentistry should not be overlooked. These include costs, maintaining quality, potential technical issues, and the accuracy of the provided information, which must be verified and approved by specialists in the field [45,76,77].

This study has several limitations. First, there is a possibility that some relevant articles were missed, despite our search criteria being designed to be as comprehensive as possible. Second, several included studies had relatively small sample sizes and short follow-up periods. Lastly, the number of studies meeting the eligibility criteria was relatively low; however, this likely reflects the availability of evidence and published research in the field.

## 5. Conclusions

Given adolescents’ and young people’s widespread access to mobile technology and the promising results reported by most authors of the selected studies, we can conclude that the field of mobile health is continuously evolving. It demonstrates clear benefits in improving oral health and hygiene behaviors and is successfully used by adolescents.

The practical relevance of this scoping review extends to dental practitioners, who can adopt digital methods for oral health education. These approaches have been proven to be more effective and engaging for children and adolescents compared to traditional methods.

This scoping review highlights the need for further long-term studies, which would enable more comprehensive evaluations, such as systematic reviews and meta-analyses, to provide a broader understanding of the effectiveness of these interventions.

## Figures and Tables

**Table 1:** Search strategies.

Database Name	Search Terms	Number of Articles Found
PubMed/Medline	teledentistry OR mHealth OR mobile application OR mobile apps OR mHealth AND adolescents OR oral hygiene AND adolescents AND mHealth, oral hygiene AND adolescents AND mobile application OR teledentistry AND mHealth	281
Scopus	teledentistry AND mHealth AND adolescents OR mHealth AND adolescents AND oral health OR mobile application AND adolescents AND oral health OR smartphone apps AND oral health AND adolescents	139
Web of Science	teledentistry AND adolescents OR mobile application AND oral health OR mHealth AND young adults AND oral health OR mobile application AND adolescents AND oral hygiene	55
Embase	adolescents AND mHealth AND oral AND health OR adolescents AND mobile application AND oral AND health OR oral AND hygiene AND teledentistry OR orthodontic AND patients AND mHealth OR preventive AND oral AND health AND adolescents AND mHealth	135
Google Scholar	teledentistry, adolescents, oral health OR mHealth, adolescents, oral hygiene OR mobile applications, adolescents, oral hygiene OR adolescents, oral prevention, mHealth OR adolescents, oral health promotion, mHealth	128

**Table 2:** Inclusion and exclusion criteria.

Criterion	Inclusion	Exclusion
Type of study	Randomized and non-randomized clinical trials	Systematic review, narrative review, scoping review, qualitative studies (focus groups)
Participants	Adolescents (aged 10–19 years)	Other age groups (under 10 years or over 19 years)
Evaluation period	Articles published between 2015 and 2024	Articles published before 2015
Availability	Full-text articles in English, with open access or institutional access	Abstract-only articles, non-open access articles, duplicates, articles published in other languages
Intervention	Use of mobile health (mHealth) technology to raise awareness of oral hygiene for preventing dental caries and gingival diseases	Irrelevant articles that do not align with the study’s objectives

**Table 3:** Characteristics of the selected studies.

Author, Year, Country	Purpose	Study Design (RCT/Non-RCT)	Sample Size	Test Group	Control Group	Follow-Up Time	Findings
Aleksejuniene, 2024, Canada [68]	To evaluate the impact of an SMS reminder strategy on adolescents’ oral self-care skills, diet knowledge, and behavior.	Non-RCT	n = 43	The adolescents (ages 11–14) received SMS reminders for oral health education following the health belief model. Oral health knowledge and oral self-care skills were assessed at the baseline (prior to education), 5 weeks (first follow-up), and 6 months (second follow-up) after the education.	No control group	6 months	Toothbrushing skill scores improved significantly (*p* < 0.001), rising from a mean of 7.0 (SD 1.9) at baseline to 14.4 (SD 2.6) at the first follow-up and 14.2 (SD 2.7) at the second follow-up. The toothbrushing time also significantly (*p* < 0.001) increased from the mean time of 51.5 (24.4) at the baseline to 112.7 (30.1) seconds at the first and to 107.2 (22.2) at the second follow-up. Diet knowledge scores improved from an average of 14.6 (4.1) at baseline to 28.0 (2.7) at the first follow-up and 25.0 (3.1) at the second follow-up. The mean sugar intake scores decreased from 12.6 (2.2) at baseline to 11.9 (1.9) at the first follow-up and 11.5 (1.9) at the second follow-up.
Alkadhi et al., 2017, Saudi Arabia [50]	To compare the impact of mobile app reminders versus verbal instructions on improving oral hygiene.	RCT	Test group n = 22 Control group n = 22	Participants received a mobile app with thrice-daily oral hygiene reminders	Participants received verbal oral hygiene instructions during routine orthodontic visits	4 weeks	Non-significant differences were found at baseline between the two groups in PI and GI values (PI = 0.8050 ± 0.4062 and GI = 0.3450 ± 0.2955 in the study group, and PI = 0.8959 ± 0.4824 and GI = 0.4927 ± 0.3005 in the control group). Significant differences were found in PI and GI (*p* = 0.04 and *p* = 0.02, respectively) between groups after 4 weeks (PI = 0.6677 ± 0.3146 and GI = 0.2273 ± 0.2256 in the study group, and PI = 0.9891 ± 0.5244 and GI = 0.5941 ± 0.5679 in the control group).
Al-Silwadi et al., 2015, UK [51]	To evaluate whether providing audiovisual information on YouTube improves orthodontic patients’ knowledge compared to traditional methods. The study also examined the effects of gender and ethnicity.	RCT	Test group n = 30 Control group n = 30	Participants (aged 13 and over) received standard verbal and written information about fixed appliances. They also received three emails over 6 weeks asking them to watch a 6-min YouTube video with similar audiovisual content. Patient knowledge was assessed using questionnaires at baseline and 6 to 8 weeks later.	Participants (aged 13+ years) received standard verbal and written information about fixed appliances.	8 weeks	The intervention group showed significantly better knowledge improvement than the control group, scoring nearly 1 point higher on average (95% CI: 0.305–1.602; *p* = 0.005). Ethnicity had a notable impact on knowledge improvement: non-Caucasian patients achieved higher scores on the second questionnaire compared to Caucasian patients, with an average difference of 0.798 points (0.158–1.438; *p* = 0.016). Gender, however, did not show a significant effect. Adding audiovisual information via the Internet to verbal and written patient information should be considered.
Al Bardaweel, Dashash, 2018, Siria [52]	To compare the effectiveness of traditional educational leaflets versus E-applications in enhancing oral health knowledge, oral hygiene, and gingival health among schoolchildren.	RCT	Test group n = 104 Control group n = 107	Schoolchildren aged 10–11 used an e-learning program for oral health education. Questionnaires were used for oral health knowledge assessment. Dental plaque was measured with PI and gingival health with GI.	Schoolchildren aged 10–11 received leaflets for oral health education.	12 weeks	At both the 6-week (*p* < 0.05) and 12-week (*p* < 0.05) intervals, the leaflet cluster demonstrated statistically significantly higher oral health knowledge compared to the e-learning cluster: the average knowledge score was 82.87 ± 10.69 at 6 weeks and 89.12 ± 8.16 at 12 weeks in the leaflet group, while the e-learning group showed values of 72.16 ± 10.25 at 6 weeks and 74.66 ± 8.98 at 12 weeks. Children in the leaflet group had significantly less plaque than those in the e-learning group at 6 weeks (PI = 1.06 ± 0.33 and 1.31 ± 0.39, respectively; *p* < 0.05) and at 12 weeks (PI = 0.85 ± 0.35 and 1.21 ± 0.40, respectively; *p* < 0.05). Children in the leaflet group had significantly better gingival health than the e-learning group at 6 weeks (GI = 0.88 ± 0.2 and 1.17 ± 0.25, respectively; *p* < 0.05) and 12 weeks (GI = 0.74 ± 0.22 and 1 ± 0.25, respectively; *p* < 0.05).
Bahaa, Selim, 2024, Egypt [53]	To examine the extent to which using a smartphone application as an educational tool and reminder of oral hygiene instructions affects patients’ oral health.	RCT	Test group n = 30 Control group n = 30	“Healthy Teeth–Tooth Brushing Reminder with timer”, an application that acts as a reminder twice a day for tooth brushing, was used in the test group (12–19 years old). GI and Quigley–Hein Turesky modified index (QHTMI) were assessed at baseline (T0) and after two months of instructions (T1).	The control group (12–19 years old) was informed of the oral hygiene instructions verbally at the baseline.	2 months	A statistically significant difference was found in the mean GI score and the mean QHTMI score of the test and control groups (*p* = 0.0002; 0.0053) respectively. While there was no statistically significant difference in the mean GI score of the oral instructions group at T0 (2.33 ± 0.66) and T1 (2.07 ± 0.69), the mean GI score of the smartphone group at T0 (2.23 ± 0.73) and T1(1.17 ± 0.74) showed a statistically significant difference. The mean score of QHTMI in the smartphone group (1.97 ± 1.03) had the most significant improvement compared to baseline (3.27 ± 1.26).
Borujeni et al., 2021, Iran [54]	To assess the impact of teledentistry on the oral health of patients with fixed orthodontic treatment during the initial and three follow-up visits.	RCT	Test group n = 30 Control group n = 30	Participants (12+ years old) who were about to begin fixed orthodontic treatment were sent an educational video via the Telegram application at baseline. PI, BOP, gingival color, and consistency were analyzed in the next three follow-up appointments.	Participants (12+ years old) who were about to begin fixed orthodontic treatment received at baseline in-person education on maintaining oral hygiene during treatment.	12 weeks	A statistically significant difference in PI and BOP was observed between the two groups at the third and fourth appointments. PI values were 32.82 ± 18.21 in the teledentistry group and 46.45 ± 22.11 in the control group. BOP values were 23.40 ± 15.73 in the teledentistry group and 36.00 ± 23.24 in the control group. However, the gingival color and consistency did not show a significant variation in relation to the method of education (*p* > 0.05). Additionally, patient age did not significantly influence oral health status in either group (*p* > 0.05).
Bowen et al., 2015, United States [55]	To study the impact of text message reminders on oral hygiene and plaque removal in adolescent orthodontic patients.	RCT	Test group n = 30 Control group n = 30	Twenty-five orthodontic adolescent patients were included in the text message group and watched, at baseline, an audiovisual presentation on how to properly brush, then received 12 messages over a period of 4 weeks, followed by 1 message per week for 8 additional weeks. Plaque was measured using planimetry. Photographs were taken at the start (T0), 4 weeks later (T1), and 12 weeks after the start (T2).	Twenty-five orthodontic adolescent patients who watched, at baseline, an audiovisual presentation on how to properly brush with a conventional toothbrush using the Bass technique	12 weeks	At T1 and T2, a significant reduction in dental plaque was observed in the test group compared to the control group. At T1, the values were 0.236 ± 0.026 for the test group and 0.438 ± 0.025 for the control group. At T2, the values were 0.221 ± 0.031 for the test group and 0.579 ± 0.030 for the control group. The conclusion was that text messages promoting good oral hygiene led to a reduction in the measurable surface area of plaque over time.
Cozzani et al., 2016, Italy [56]	To assess the impact of structured follow-up communication after the application of orthodontic appliances on oral hygiene adherence in 30–40 days.	RCT	Test group 1 n = 28 Test group 2 n = 26 Control group n = 30	Among orthodontic patients (age 10–19 years), the first test group received a reassuring structured text message, and the second test group received a structured phone call to encourage appropriate dental hygiene 5–7 h after bonding. The Plaque Index was measured for all patients.	Orthodontic patients (age 10–19 years) who received no post-procedure communication	30–40 days	Participants who received post-treatment communication showed higher oral hygiene compliance than those in the control group, with plaque indices of 0.3 and 0.75, respectively (*p* = 0.0205). Follow-up after orthodontic treatment may effectively enhance short-term oral hygiene compliance.
Deleuse et al., 2020, Belgium [57]	To compare the effectiveness of a standard oscillating/rotating electric toothbrush to an interactive version connected to a brushing aid app in adolescents with fixed orthodontic appliances.	RCT	Test group n = 19 Control group n = 19	Adolescents (12–18 years) with full-fixed orthodontic appliances used an interactive oscillating/rotating electric toothbrush connected to a brushing aid app. At baseline, all patients received verbal and written oral hygiene instructions. Patients were evaluated for PI, GI, and WSL from baseline (T1) to the end of the study (T4).	Adolescents (12–18 years) with full-fixed orthodontic appliances used an oscillating/rotating electric toothbrush alone. At baseline, all patients received verbal and written oral hygiene instructions.	18 weeks	From T1 to T4, the PI significantly decreased in both groups (control: *p* < 0.0001; test: *p* = 0.0003), with no significant difference between them. Similarly, the GI significantly decreased in both groups (control: *p* = 0.0028; test: *p* = 0.0008) and showed no difference between them. WSL scores remained stable in both groups (control: *p* = 0.066; test: *p* = 0.73) with no significant difference (*p* = 0.28).
Fageeh et al., 2024, Saudi Arabia [58]	To evaluate the effectiveness of the Telesmile mobile app in improving oral health knowledge and hygiene practices among individuals who were blind and deaf.	RCT	Test group n = 50 Control group n = 50	Participants aged 12–18 (blind n = 25, deaf n = 25) used the Telesmile app, featuring multimedia Arabic dental sign language videos for patients with deafness and audio-recorded oral hygiene instructions for patients with blindness. Participants’ knowledge was assessed using a close-ended questionnaire at the initial visit (T0) and after 4 weeks (T1).	Subjects aged 12–18 (blind n = 25, deaf n = 25) received regular oral hygiene instructions.	4 weeks	Initially, participants who were blind and deaf had poor knowledge of oral health and hygiene (T0). After using the Telesmile app for 4 weeks (T1), their knowledge significantly improved (*p*< 0.001). Audio techniques effectively educated blind participants, while video demonstrations enhanced the oral health knowledge of deaf individuals.
Farhadifard et al., 2020, Iran [59]	To evaluate the effectiveness of the Brush DJ smartphone application in promoting oral hygiene adherence among patients with fixed orthodontic appliances.	RCT	Test group n = 60 Control group n = 60	Patients aged 15–25 starting fixed orthodontic treatment received conventional oral hygiene instruction, then used the Brush DJ app. PI and GI were measured at baseline (T0), and at 4, 8, and 12 weeks (T3). Tooth brushing frequency and duration were also recorded.	Patients aged 15–25 starting fixed orthodontic treatment received conventional oral hygiene instruction.	12 weeks	PI and GI improved in the intervention group but worsened in the control group, showing significant differences (*p* < 0.001). PI decreased from T0 = 75.21 ± 13.36 to T3 = 67.84 ± 12.33 in the interventional group and increased from T0 = 76.59 ± 12.76 to T3 = 80.82 ± 10.5 in the control group. GI drcreased from T0 = 1.29 ± 0.49 to T3 = 0.95 ± 0.43 in the intervention group and from T0 = 1.49 ± 0.59 to T3 = 1.43 ± 0.56 in the control group. Increased brushing frequency and duration were linked to app usage during follow-up.
Innes et al., 2024, UK [60]	To evaluate the effectiveness of promoting toothbrushing to prevent dental caries (Brushing RemInder 4 Good oral HealTh, BRIGHT) in UK secondary schools.	RCT	Test group n = 2262 Control group n = 2418	Pupils aged 11–13 who owned mobile phones and attended secondary schools with high free meal eligibility received a lesson and twice-daily texts.	Pupils aged 11–13 who received routine education	2.5 years	At 2.5 years, no significant difference in caries prevalence was observed. Twice-daily toothbrushing rates initially reported by 77.6% of pupils increased at 6 months (intervention: 86.9%; control: 83.0%; OR 1.30, 95% CI 1.03–1.63, *p* = 0 .03) but showed no difference at 2.5 years (intervention: 81.0%; control: 79.9%; OR 1.05, 95% CI 0.84–1.30, *p* = 0.69).
Krishnan et al., 2021, India [69]	To assess the impact of two sensory-based interventions—visual pedagogy and the mobile app Brush Up—on improving oral health in adolescents with Autism Spectrum Disorder.	Non-RCT	Visual pedagogy group n = 30 Brush Up - mobile application group n = 30	Children with ASD aged 13–17 years used the Brush Up mobile application, based on oral health education, with an interactive toothbrush training game in it. Plaque Index and Gingival Index were assessed at baseline, after the 6th week, and after the 12th week.	Children with ASD aged 13–17 years received visual pedagogy—visual cards depicting toothbrushing technique, to be taken at home.	12 weeks	Significant differences in plaque (*p* < 0.001) and gingival scores (*p* < 0.001) were observed among groups at 6 and 12 weeks after intervention. No significant differences in dental plaque (*p* = 0.912; 1.023; 0.812) or gingival scores (*p* = 0.932; 0.264; 0.283) were noted between the groups at any timepoint: PI = 2.02, 1.00, and 0.45; GI = 1.05, 0.61, and 0.28 in visual pedagogy group; PI = 2.02, 1.00, and 0.46; GI = 1.03, 0.58, and 0.24 in Brush Up group.
Kumar et al., 2022, India [61]	To compare the effects of an interactive game-based visual performance technique (IGVP) and traditional oral health education (OHE) on plaque control, gingival health, and knowledge and practices of oral hygiene.	RCT	Test group n = 50 Control group n = 50	Children aged 12–15 received the IGVP technique.	Children aged 12–15 received traditional OHE	3 months	The test group had a 58.7% reduction in gingival scores and a 63.4% reduction in plaque scores after intervention compared to the control group, which showed 2.8% and 0.7% reductions (*p* < 0.001). The test group also gained 22.4% more knowledge, while the control group gained 7.8%.
Lopes Dos Santos et al., 2023, Brazil [62]	To assess the impact of a mobile app on the oral hygiene (OH) of adolescents with fixed orthodontic appliances.	RCT	Test group n = 4 Control group n = 4	Adolescents (14–19 years old) received OH guidance and motivation via a custom app. Clinical assessments using VPI and GBI were conducted at five time points: before orthodontic device application (T0); at baseline (T1); and 30 (T2), 60 (T3), and 90 (T4) days after.	Adolescents (14–19 years old) received standard OH instructions.	90 days	Although no significant difference could be observed, VPI at T1 and T2 were lower for volunteers in the experimental group (33.20 ± 19.29; 32.10 ± 7.72) than for the volunteers in the control group (42.11 ± 8.56; 43.59 ± 34.71). The same was observed for GBI, in which volunteers in the experimental group presented lower GBI at T1 and T2 (12.70 ± 8.10; 13.72 ± 7.39) than volunteers in the control group (27.53 ± 17.89; 20.38 ± 9.95). Good acceptance for using the app was shown by volunteers.
Marchetti et al., 2018, Brazil [63]	To assess the effectiveness of a mobile oral health app associated with conventional educational methods in improving adolescents’ periodontal health	RCT	Test group n = 141 Control group n = 147	Adolescents aged 14–19 years received oral guidance (OG) on oral health maintenance. Two sub-groups were created: one sub-group received access to a mobile oral health app where reinforcement messages were sent twice a day for a period of 30 days, and the other did not use the app. The knowledge score (KS) on oral health, OHI-S, and GBI were assessed at baseline and 20 weeks after.	Adolescents aged 14–19 years received video guidance (VG) on oral health maintenance. Two sub-groups were created: one sub-group received access to a mobile oral health app where reinforcement messages were sent twice a day for a period of 30 days, and the other did not use the app.	20 weeks	A significant difference was observed in the KS mean in the follow-up test among the adolescents who used the app (mean = 4.77 ± 0.52) and those who did not have access to this educational method (4.35 ± 0.66; *p* < 0.001), regardless of the type of previous intervention (OG or VG). OHI-S and GBI decreased significantly (*p* < 0.001) in all groups, with no significant differences between groups. The app was effective in increasing knowledge, especially associated with video. The different methods were equally effective for a better standard of oral hygiene.
Santos et al., 2021, Brazil [64]	To evaluate how WhatsApp text messages affect patient awareness of daily oral hygiene and flossing importance.	RCT	Test group n = 22 Control group n = 22	Patients with a mean age of 14.3 ± 2.6 years with fixed orthodontic appliances received instructions on toothbrushing and flossing and daily WhatsApp text messages. PI, GBI, and halitosis were assessed at baseline and after 30 days.	Patients with a mean age of 14.3 ± 2.6 years with fixed orthodontic appliances received instructions on toothbrushing and flossing only, with no text messages.	30 days	Flossing frequency increased in both groups. In the test group, the frequency rose by 59.1% (*p* < 0.001), significantly higher than the control group (*p* > 0.05). The control group showed a 31.8% rise in frequency (*p* < 0.03). PI and GBI significantly decreased over time in both groups; PI decreased from 3.0 to 2.0 in the test group and from 2.5 to 2.0 in the control group, and GBI decreased from 3.0 to 2.0 in both groups (*p* < 0.05). In the test group, the halitosis score decreased significantly (*p* < 0.05)
Scheerman et al., 2020, Iran [65]	To assess the effectiveness of the WhiteTeeth mobile app, a theory-based mHealth program for oral hygiene improvement in adolescent orthodontic patients.	RCT	Test group n = 67 Control group n = 65	Adolescent orthodontic patients received the WhiteTeeth app alongside usual care. Data (Plaque Index, bleeding on probing index, and a questionnaire on oral health behavior) were collected at three check-ups: baseline (T0), 6 weeks (T1), and 12 weeks (T2).	Adolescent orthodontic patients received usual care only	12 weeks	At 6 weeks, the intervention significantly reduced gingival bleeding (B = −3.74; 95% CI −6.84 to −0.65) and increased fluoride mouth rinse use (B = 1.93; 95% CI 0.36 to 3.50). At 12 weeks, the intervention group showed a greater reduction in dental plaque accumulation (B = −11.32; 95% CI −20.57 to −2.07) and in the number of plaque-covered sites (B = −6.77; 95% CI −11.67 to −1.87) compared to the control group.
Scheerman et al., 2020, Iran [66]	To test the effectiveness of a theory-based program using Telegram (an online social media platform) to promote oral hygiene among Iranian teenagers.	RCT	Group A n = 253 GroupA + M n = 260 Control group n = 278	Group A: Telegram group with adolescents (12–17 years) only. Group A + M: Telegram group with adolescents and mothers. Periodontal condition and plaque status were assessed by two trained dental professionals using the VPI and CPI.	Adolescents (12–17 years) did not receive any intervention during the experimental phase of the intervention.	6 months	At 1- and 6-month follow-ups, toothbrushing frequency was significantly higher in both intervention groups compared to the control. The A + M group showed greater improvements in toothbrushing, VPI (from 2.71 to 2.12), and CPI (from 1.68 to 1.33) scores than the A group. At 6 months, the A + M group also reported significantly more perceived social support from mothers than the A and control groups.
Zahid et al., 2020, Saudi Arabia [70]	To assess the impact of two oral health education approaches—the Brush DJ mobile app and conventional lectures—on high-school children’s oral hygiene knowledge and behavior.	Non-RCT	Test group n = 130 Control group n = 141	The mobile app group used the app twice daily for 3 months.	The conventional education group received a 20 min lecture on oral hygiene using a whiteboard, markers, slides, and dental models.	3 months	The Brush DJ app was as effective as educational lectures in improving oral health knowledge, attitude, and behavior. Both groups showed significant improvements in most areas, except for the app group’s toothbrushing frequency and duration. No change was seen in twice-daily brushing among app users, and less than 40% brushed for 2 min. In contrast, the lecture group showed significant improvement in these areas: the percentage of subjects reporting twice-daily brushing increased from 52.5% to 75.0%. The app was also found to be more difficult to use than the lectures (*p* = 0.037).
Zotti et al., 2016, Italy [67]	To test the effectiveness of an app-based protocol for oral hygiene maintenance in improving compliance and oral health in adolescents with fixed multibracket appliances.	RCT	Test group n = 40 Control group n = 40	Adolescents starting orthodontic multibracket treatment received standardized oral hygiene instructions at baseline and were enrolled in a WhatsApp chat room-based competition and instructed to share monthly two self- photographs (selfies) with the other participants showing their oral hygiene status. PI, GI, WSL, and caries presence were recorded for all patients.	Adolescents starting orthodontic multibracket treatment received standardized oral hygiene instructions at baseline.	12 months	After 6, 9, and 12 months, the study group had significantly lower PI and GI scores and a reduced incidence of new WSL compared to the control group. PI for the control group at T0 was 0.48(0.34) and at T4 was 1.79(0.54), and PI for the study group at T0 was 0.41 (0.32) and at T4 was 1.06(0.47). GI for the control group at T0 was 1.17(0.66) and at T4 was 1.40(0.57), and GI for the study group at T0 was 1.18(0.67) and at T4 was 0.67(0.36). The number of patients with WSL was 4(T0) and 7 (T4) in the study group and 5 (T0) and 16 (T4) in the control group.

RCT = Randomized Controlled Trial; PI = Plaque Index; GI = Gingival Index; BOP = Bleeding on Probing; WSL = White Spot Lesion; GBI = Gingival Bleeding Index; VPI= Visible Plaque Index; CPI = Community Periodontal Index; QHTMI = Quigley–Hein Turesky modified Index; SD = Standard Deviation; OHI-S = Simplified Oral Hygiene Index.

**Table 4:** Cochrane risk of bias of randomized controlled trials (RoB2) (green symbol = low risk of bias, yellow symbol = high risk of bias, and blue symbol = concern).

	Bias Arising from the Randomization Process	Bias Due to Deviations From Intended Interventions	Bias Due to Missing Outcome Data	Bias in Measurement of the Outcome	Bias in Selection of the Reported Result	Overall
Alkadhi et al., 2017 [50]	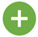	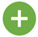	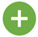	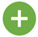	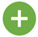	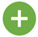
Al-Silwadi et al., 2015 [51]	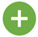	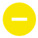	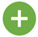	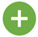	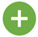	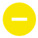
Al Bardaweel, Dashash, 2018 [52]	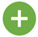	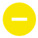	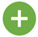	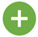	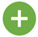	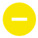
Bahaa, Selim, 2024 [53]	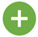	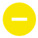	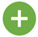	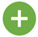	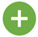	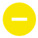
Borujeni et al., 2021 [54]	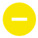	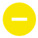	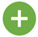	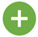	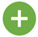	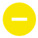
Bowen et al., 2015 [55]	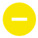	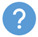	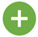	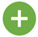	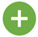	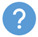
Cozzani et al., 2016 [56]	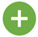	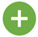	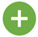	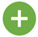	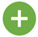	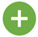
Deleuse et al., 2020 [57]	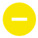	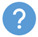	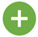	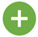	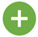	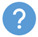
Fageeh et al., 2024 [58]	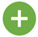	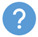	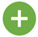	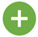	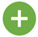	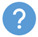
Farhadifard et al., 2020 [59]	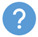	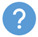	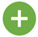	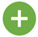	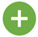	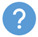
Innes et al., 2024 [60]	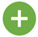	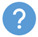	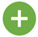	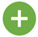	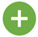	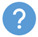
Kumar et al., 2022 [61]	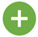	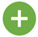	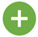	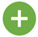	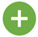	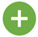
Lopes Dos Santos et al., 2023 [62]	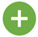	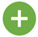	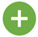	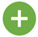	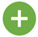	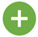
Marchetti et al., 2018 [63]	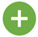	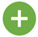	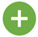	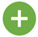	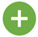	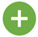
Santos et al., 2021 [64]	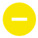	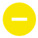	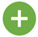	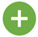	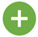	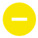
Scheerman et al., 2020 [65]	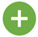	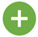	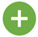	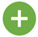	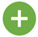	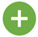
Scheerman et al., 2020 [66]	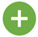	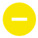	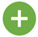	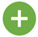	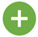	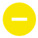
Zotti et al., 2016 [67]	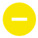	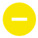	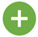	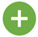	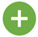	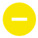

**Table 5:** Risk of bias for non-randomized controlled trials (ROBINS-I) (green symbol = low risk of bias, yellow symbol = serious risk of bias, and blue symbol = moderate risk of bias).

	D1	D2	D3	D4	D5	D6	D7	Overall
Aleksejuniene, 2024 [68]	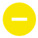	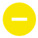	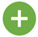	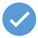	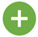	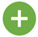	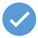	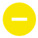
Krishnan et al., 2021 [69]	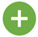	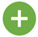	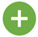	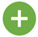	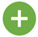	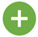	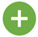	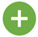
Zahid et al., 2020 [70]	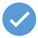	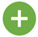	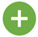	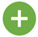	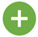	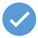	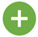	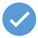

D1: Risk of bias due to confounding; D2: Risk of bias in classification of interventions; D3: Risk of bias in selection of participants into the study (or into the analysis); D4: Risk of bias due to deviations from intended interventions; D5: Risk of bias due to missing data; D6: Risk of bias arising from measurement of the outcome; D7: Risk of bias in selection of the reported result.

## Data Availability

The data that support the findings of this study are available upon request from the corresponding author.

## Supplementary Materials

The following supporting information can be downloaded at: https://www.mdpi.com/article/10.3390/jcm14092907/s1, Table S1: Scoping Reviews (PRISMA-ScR) Checklist.
